# The Impact of Job Demands, Job Resources, and Organisational Justice on Global Health and Turnover Intentions in Animal Care Workers

**DOI:** 10.3390/ani15030420

**Published:** 2025-02-03

**Authors:** Remi Lezon, Vanessa Rohlf, Diana Rayment, Tiffani J. Howell

**Affiliations:** 1 School of Psychology and Public Health, La Trobe University, Bundoora, VIC 3086, Australia; and Bendigo, VIC 3552, Australia; 21011189@students.latrobe.edu.au (R.L.); v.rohlf@latrobe.edu.au (V.R.); 2 PetRescue Ltd., Perth, WA 6000, Australia; diana.rayment@petrescue.org.au

**Keywords:** workplace health and safety, wellbeing, animal caregiver

## Abstract

Animal care workers in sheltering, rescue, and management are exposed to occupational stressors which negatively impact health. While the negative mental health impacts have been previously documented in this population, physical health, and its contributing factors, have not. This study investigated how job demands, job resources, and organisational justice relate to mental and physical health, and how they subsequently affect turnover intentions. Of the 285 participants, aged 19 to 94 years, who completed the online anonymous questionnaire, below average mental and physical health was reported. Mental health, but not physical health, was inversely related to intentions to leave. After controlling for age, hierarchical multiple regressions showed high emotional demands and direct euthanasia exposure significantly predicted poorer mental and physical health, while high levels of social support predicted better mental and physical health. No additional variance in either health domain was accounted for by organisational justice. It was concluded that both physical and mental health may be negatively impacted in those who work in shelter, rescue, and management environments which could lead to high turnover and poor outcomes for individuals and organisations. Monitoring through ongoing health records to enable early intervention and accommodations should be considered to promote the health of these workers.

## Simple Summary

Animal care workers in sheltering, rescue, and management experience workplace stressors which negatively impact health, and as a result, increase their likelihood to leave their role. Existing research has explored several mental health and wellbeing ailments, yet few consider physical health. Using a questionnaire disseminated to Australian employees and volunteers working in the sector, we found emotionally challenging situations like participation in animal euthanasia, coupled with a lack of perceived social support, were related to poorer mental and physical health. Poorer mental health was weakly linked to a higher likelihood of leaving one’s role or the field. How fairly respondents felt they were treated by their organisation did not appear to impact health outcomes, after considering the effects of other work-related factors. Its impact on health should not be ruled out entirely, however, and requires further research. Based on current findings, to protect the health of these workers, and subsequently to minimise staff turnover, we suggest rotating tasks assigned to workers to lessen the frequency of engagement with demanding tasks, while still allowing for essential or unavoidable tasks to be completed. Routine monitoring of staff and volunteer health is also recommended to identify at-risk individuals to enable early intervention and accommodations.

## 1. Introduction

Those in professions which require caring for others are united by their empathy and compassion for those who need it. These care professionals may include nurses, psychologists, veterinarians, and animal shelter workers, or informal caregivers, i.e., those who care for sick relatives or pets. These roles share similar demanding tasks, such as providing a consistent level of empathy for those that are suffering or unwell, and these demands can pose a risk to the health and wellbeing of care workers [1,2]. Declining wellbeing amongst those in caring roles also has repercussions for the recipients of their care, as they may face poorer outcomes as a result [3].

Much existing research focuses on the mental impact of these demands in those who care for people [4,5], through concepts such as compassion fatigue in professionals and ‘caregiver burden’ in informal care workers. Compassion fatigue is conceptualised as an emotional state of exhaustion and diminished capacity to care [6,7,8] as the result of empathetic exposure to the trauma of others [9] and burnout caused by persistent high occupational stress [10,11,12]. ‘Caregiver burden’ [13] also explores how the time, physical, and emotional commitments involved in care provision can negatively impact the emotional and social functioning of those in informal caring roles [14]. Naturally, however, these conditions and challenges are not exclusive to those who care for people, and there are clear indications that animal care workers also indicate stress in response to their work [15,16].

Animal care workers vary in their roles and responsibilities, with many being volunteers who may maintain another job, or are retired [17]. These factors can make it challenging to determine who and how these workers are affected, as they may not have recorded employment, or their role may be filtered into different categories. Current research into the wellbeing of animal care workers has highlighted difficulties such as burnout [18], grief [19,20], compassion fatigue [21,22], depression, and sleeplessness [23,24] to be prevalent in these roles. Further, veterinarians report a likelihood of suicide four times greater than the general population [25,26]. Studies that investigate the wellbeing of animal care workers often adopt concepts and strategies created for other caring professions out of necessity, such as compassion fatigue, and while this is important in determining the impact of caring work across disciplines, animal care workers take on a number of additional demands unique to their sector that may expand how they are impacted.

Animal care workers face several unique challenges, such as tending to injured and traumatised animals, comforting grieving owners, and euthanising animals they have had a role in caring for [27,28,29]. Animal care workers in shelters and rescues are noted for their high empathy for animals and work in these roles to protect unwell and homeless animals [30]. This moral motivation, in addition to their attachment to animals, can lead to their continued work, despite feelings of distress from their role [31]. Further, it is also common for those in shelters or foster care positions to bring their work home with them [31], reducing time for self-care and increasing attachment to potentially stressful situations. Accordingly, high occupational stress is reported by both shelter employees and volunteers [21], such that one survey of 1000 shelter and animal-control workers by the Humane Society of the United States, reported in Figley and Roop [6], found 56.4% of the sample self-reported as extremely high risk for compassion fatigue. Thus, shelter and rescue workers may be particularly susceptible to the challenges of animal care work.

Euthanasia exposure is often also assumed to play a unique role in diminishing animal care worker wellbeing, but research in this area is conflicting. Participation in euthanasia has been linked to poorer mental health in animal shelter workers by Baran et al. [32] and Scotney et al. [15]. Other studies, however, found that neither indirect nor direct exposure to euthanasia had a significant impact on wellbeing [19,33,34]. Conflicting findings may be due to factors such as the relationship between the care worker and the animal [35], the reason for euthanasia [36], whether one morally agrees with the decision [37], or whether they feel responsible for it [19]. More research is needed to understand this variability relating to the impact of euthanasia in shelter and rescue workers.

Organisationally, poor wellbeing amongst animal care workers can increase intentions to leave their role or the field altogether [38,39,40,41], and this subsequent turnover is notably highest within a year of first exposure to animal euthanasia [42,43]. This can increase work pressure on remaining staff [44] and interfere with organisational processes [45].

It is clear that the mental health fallout of work stress is central in available animal care worker literature [20]. While this research is vital in identifying areas which can be targeted in wellbeing interventions, the interrelationships with other domains of health have been understudied in this population. Health is a complete sense of wellbeing across physical, mental, and social factors [46]. The limited research on other health domains is despite findings that link burnout with physical fatigue and pain in other contexts such as teaching, social work, and nursing [47], as well as increased physiological indicators such as blood pressure and heart rate being linked with the high stress in animal shelter and veterinary roles [19,34]. Reeve et al. [42] also highlighted how poor physical wellbeing in animal care workers may occupy greater organisational resources. Additional research which adopts a wholistic view of health, and its contributing factors, in this subset of workers is required.

A potential way to conceptualise the relationship between health and occupational factors in animal care workers is through the Job Demands–Resources (JD-R) framework [48]. This framework identifies all aspects of a job as either demands or resources. Job demands are those with a physical, psychological, social, cognitive, or emotional cost to the worker [48], and within the model include work pressure and emotional and cognitive demands [49]. Job resources aid in the completion of work, reduce work demands, or promote growth [48]. Some examples include social support, feedback, and autonomy [49]. Resources have been linked with improved wellbeing, improved job satisfaction, and lower turnover [50]. Some challenges animal care workers face that contribute to demands within the model, include: grief [20]; workload or job insecurity [51]; and, although unique, animal euthanasia exposure [15]. Social and organisational support [40,52] and empathy for animals [20,53] are considered notable resources. When resources cannot mitigate demands, continued work may pose a risk to the health of care workers.

Monaghan et al. [33] utilised the JD-R to examine wellbeing in employees and volunteer animal rescue and shelter facilities, finding that significant traumatic stress and burnout variance was explained by job demands, but job resources did not moderate this relationship, contrary to expected JD-R relationships [50]. Instead of moderation, studies examining the capability of resources in directly predicting wellbeing in veterinarians and shelter workers provide better support for the JD-R in this context [40,54], although the impact of different resources varies across studies. One study by Paul et al. [20] examined how JD-R factors predict wellbeing in animal care workers, finding that both the severity of their grief experience and perception of organisational support were significant, opposing predictors of burnout. The authors’ qualitative findings suggested further research exploring organisational resources is necessary, as participants’ wellbeing concerns arose from factors predominantly under organisational control.

Organisational justice (OJ) presents one resource that may be important to animal care workers in shelters and rescues. OJ represents an employee’s perception of fairness within an organisation, split across four justice dimensions: distributive, procedural, interpersonal, and informational [55,56]. These dimensions refer to the degree to which employees perceive outcomes are a fair reflection of their input (i.e., distributive), that workplace procedures are fair and consistent (i.e., procedural), that they are treated respectfully by authority (i.e., interpersonal), and their belief that there have been adequate communications from superiors (i.e., informational) [55,56]. High OJ perceptions in healthcare have predicted better work engagement, better wellbeing, and lower turnover [57,58], whereas lower OJ perception in non-healthcare work environments has been linked with psychological distress [59], poor self-rated health [60], and higher risk of sickness-related absence [61]. Not yet examined in this population, there is partial support for the notion that OJ is important for animal care workers as studies of similar concepts such as ‘voice’ [17], job control [52], and decision-latitude [23] found that these play a role in their satisfaction and wellbeing. If this is the case, further research specifically exploring the role of OJ in this population is necessary.

**Figure 1:**
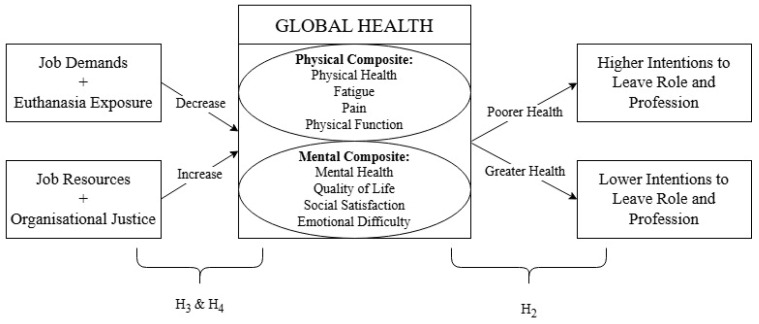
Possible relationships between job demands–resources, organisational justice, health, and turnover intentions. Note: H_1_ is not evaluating a relationship between variables but rather a current view of health within the target population and thus was not included in the figure above.

The health of animal care workers in shelters, rescues, and management facilities (e.g., municipal pounds) may be more broadly affected by their work than current literature indicates, as elements of health, such as physical health, have not been explored to the same extent as mental wellbeing, despite evidence suggesting they may be affected. It would be instructive to examine wider health factors in these animal care workers and explore contributing factors using the JD-R, including the role of OJ. As such, this study aimed to explore global health among those working in animal management, sheltering, and rescue, as well as to assess whether this is associated with turnover from work, job demands and resources, and OJ. First, it was hypothesised that participants would self-report lower than average physical and mental health (H_1_). Second, self-reported health would be inversely correlated with turnover intention (H_2_). Third, higher job demands would predict poorer self-reported health, whilst higher resources would predict greater health (H_3_). Fourth, higher OJ perception would predict greater health (H_4_). The explored relationships are shown in Figure 1.

## 2. Materials and Methods

This study was part of a larger collaborative project with PetRescue, a national charity that facilitates the adoption of pet animals, coordinating with other rescue organisations in Australia. Only survey items related to the present study aims are detailed.

This study was approved by the Human Research Ethics Committee of La Trobe University (HEC24226). Individuals working within the sector were consulted during question design to ensure relevant topics were addressed and question wording was suitable and appropriate to those working across the sector. Participants were fully informed of the study aims and were provided with contact details of support services (i.e., Lifeline and Beyond Blue) to access should they experience discomfort and want to talk to someone. This information was presented in a Participant Information and Consent Form, and again at the beginning and towards the end of the survey.

### 2.1. Participants

A snowball sample of animal care workers was recruited between July and August 2024. It was required that participants were: currently residing in Australia; over the age of 18 years old; able to read and write in English, the language of the survey; and, working in a paid or volunteer role for an animal management, shelter, and/or rescue facility. A link containing recruitment, study, and consent information was distributed to animal shelters in the rescue directory of PetRescue via email, advertised on social media (i.e., Facebook, LinkedIn) through PetRescue and other state and national peak bodies, as well as shared within research groups. Participants were also asked to forward the study to relevant industry contacts. A total of 406 responses were recorded. Those who did not attempt the full battery of health and workplace measures were excluded, although demographic items were optional and full completion of these was not required for inclusion. The final sample included 285 participants with usable questionnaire completion. Participants ranged from 19 to 94 years of age (M = 49.8, SD = 15.6). The wide age range in the sample may be explained by the prevalence of volunteers within this industry, and thus it may have attracted those outside typical working age or circumstance (e.g., those that are retired from full-time work).

### 2.2. Measures

The present study utilised a cross-sectional survey design. Data collection was conducted using an online anonymous survey hosted on QuestionPro Survey Software [62].

#### 2.2.1. Demographics

Individual and organisation demographic factors were recorded using 9 items developed by the research team in consultation with industry members. Participants were asked for role and organisation information, in addition to their age and gender. Participants could select multiple roles as this is not uncommon in this field [63]. It is notable that significantly fewer responses for the item relating to the participant’s gender identity were recorded. During consultation, it was indicated by some industry members that they felt queries of gender may not seem relevant to all respondents when answering a survey about health and their experiences in the industry. This, together with the item being optional and positioned near the conclusion of the survey, may explain the lower response numbers in comparison to other demographic items. Table 1 details demographic characteristics for the final sample of animal management, shelter, and rescue workers that participated in the study.

#### 2.2.2. Global Health

The Patient-Reported Outcomes Measurement Information System—Global Health v1.2 10-item scale (PROMIS-GH; [64] measured self-reported global health, where every item considers a different domain of health [65]. Six PROMIS-10 items are scored on a 5-point Likert scale from poor to excellent health, one item addressing physical ability is scored from ‘not at all’ to ‘completely’, two items referring to emotional difficulties and fatigue are displayed negatively where higher values indicate worse health, and one item addresses level of pain on a 10-point scale from ‘no pain’ to ‘worst pain imaginable’ that is recoded during analysis to a 5-point response. Usage of the PROMIS-GH was in adherence with the Terms of Use for research publications outlined by HealthMeasures.

Four items comprise a physical health composite (PCS; i.e., health, functioning, pain, fatigue, e.g., “To what extent are you able to carry out your everyday physical activities such as walking, climbing stairs, carrying groceries, or moving a chair?”), and four items correspond to a mental health composite (MCS) that is inclusive of social factors (i.e., quality of life, mental health, social satisfaction, emotions, e.g., “In general, how would you rate your mental health, including your mood and your ability to think?”). Two individual items (i.e., general health and social functioning) are not used in the composites; however, they are highly correlated with PCS and MCS [65].

Consistent with previous studies [66,67], only the composites were used for analysis as individual health items are not as precise [68]. PCS and MCS were converted to t-scores included with the measure, with a mean of 50 and SD of 10 based on general population norms [69]. Composite scores of the PROMIS-GH have good internal reliability (MCS, α = 0.88; PCS, α = 0.82) and support for convergent and divergent validity in previous research (Cella et al., 2010). PROMIS-GH is primarily used with inpatients, although it has previously been used to study the health of unpaid cancer caregivers [67].

#### 2.2.3. Intentions to Leave

Intentions to leave were measured with two items [33,54] rated on 5-point Likert scales from ‘not at all likely’ to ‘extremely likely’, asking participants to report their likelihood to leave their current role, and animal caregiving work entirely, within the next 12 months. Turnover intention is considered a reliable predictor of actual turnover [70,71].

#### 2.2.4. Job Demands and Resources

The Job Demands-Resources Questionnaire (JD-RQ; [49]) was used to measure job demands and resources. The present study included job demand scales of work pressure, cognitive demands, and emotional demands, as well as job resource scales of autonomy, social support, and feedback, totalling 20 items. Participants rated items on a 5-point Likert scale from ‘never’ to ‘very often’, or ‘strongly disagree’ to ‘strongly agree’, where high scores correspond with high perceived demands or resources. Reliability and validity for subscales are not available in Bakker et al. [49], but this scale has been widely used to measure job demands and resources, and it has been shown to be reliable (JD, α = 0.82; JR, α = 0.76) in previous, similar research [27,33].

#### 2.2.5. Euthanasia Exposure

Euthanasia exposure was measured using two items [33,54] that assess direct (participation, e.g., “To what extent are you directly involved in the euthanasia of animals you care for (e.g., restraining the animal, injecting the solution)?”) and indirect (knowledge of events, witnessing, e.g., “To what extent do you have knowledge of animals you have cared for being euthanised, even when you do not participate directly in the procedure?”) exposure. Items were scored on 5-point Likert scales from ‘not at all’ to ‘completely’, where higher scores indicated greater exposure.

#### 2.2.6. Organisational Justice

A shortened version of Colquitt’s [55] 20-item, four-dimension organisational justice scale, reduced to eight items and three dimensions by Elovainio et al. [72], was used to measure perceptions of OJ. The shortened scale was chosen to reduce the time participants needed to complete the full test battery. Items addressed procedural justice, distributive justice and interpersonal justice. The shortened scale excluded the informational justice dimension, as items overlapped with constructs captured more reliably by interpersonal justice items [72]. Participants responded on a 5-point Likert scale ranging from ‘to a small extent’ to ‘to a large extent’, where higher scores indicated higher perceived OJ. Certain items require adjustment depending on the context in which the scale is used, e.g., “My [outcome] is appropriate for the work I complete” [73]. Animal care workers may feel a number of benefits from their work, such as a sense of fulfilment, beyond how they are compensated or treated, and thus items were adjusted to allow for this interpretation, e.g., “The benefits I get from work are appropriate for the work I complete”. The shortened scale has good internal consistency, structural validity, and predictive validity [72] and the original scale has been used to examine turnover amongst nurses [58].

### 2.3. Procedure

The questionnaire, inclusive of items from the larger study, took an average of 29 min to complete. Participants read an information statement informing them of the questionnaire contents, that their responses after supplying consent would be recorded, and that they could withdraw from further responses at any time. Once participants selected ‘I agree, start questionnaire’, they were presented with demographic questions followed by the study measures.

### 2.4. Analyses

Analyses were conducted using IBM SPSS Statistics for Windows, Version 29.0.2.0 [74]. Frequencies and descriptives identified the sample, provided reliability of subscales, and evaluated the overall health levels reported, addressing H_1_. Normality of dependent health variables was assessed through Kolmogorov–Smirnov tests, as well as evaluation of histograms and box plots. Kolmogorov–Smirnov statistics for PCS and MCS were significant (*p* < 0.001), suggesting a violation of the assumption of normality [75]; however, the large sample size indicated parametric tests were still suitable for analysis [76]. Pearson’s correlations were then calculated to test the relationships that MCS and PCS had with turnover intention, addressing H_2_, in addition to euthanasia exposure, job demands, job resources, OJ, and age to examine zero-order relationships these predictors had with health outcomes.

To increase regression power, predictors were excluded if their correlation with health outcomes was non-significant [77]. Hierarchical multiple regressions predicting each of MCS and PCS were then run. Assumptions of normality, linearity, and homoscedasticity for each regression were examined using normal probability plots of the regression of standardised residuals, as well as scatterplots of standardised residuals against standardised predicted values. Correlation matrices and tolerance values were checked to assess multicollinearity amongst predictors. Multivariate outlier presence was assessed through Mahalanobis distances by comparing these to the critical value for the number of predictors in the regression, calculated using an alpha level of 0.001 [78].

Age was entered at step 1 of the regressions to control for confounding effects on health. Direct and indirect euthanasia exposure were entered at step 2 to evaluate these separately from other demands. Job demands and job resources subscales were entered at step 3 to assess their relationship with health, addressing H_3_. Finally, perceptions of OJ dimensions were entered at step 4 to see whether this predicted additional variance in health, after controlling for other known factors, per H_4_.

## 3. Results

### 3.1. Overview of Scales and Reliability Analysis

The means and standard deviations for scales of health, intentions to leave, job demands, job resources, euthanasia exposure, and OJ are available in Table 2. Further, Table 2 contains the internal reliability analyses for the PROMIS-GH composite subscales, as well as both overall and subscale internal reliability for the JD-R and OJ scales. All subscales had Cronbach α above 0.70, indicating good reliability [75].

### 3.2. Health of Animal Care Workers in Shelter, Rescue, and Management Facilities

Relative to normative data (M = 50, SD = 10), physical health (M = 43.9, SD = 7.7, 95% CI [35.4, 52.5]), and mental health (M = 42.1, SD = 9.1, 95% CI [34.8, 49.4]) were lower than average across the sample, indicating poorer health. PCS t-scores < 42 and MCS t-scores < 40 indicate fair to poor health [79,80]. MCS in 42.8% (*N* = 122) of participants, and PCS in 32.2% (*N* = 92) of participants, fell below these cutoffs. Nonetheless, the mean scores fell into the range of good health on domains, despite being lower than the average of the normative data.

### 3.3. Intentions to Leave in Animal Shelter, Rescue, and Management

Participants’ intention to leave their current role in animal care work, as well as intention to leave the profession altogether, in the next 12 months are shown in Table 2. Mean intentions to leave current role and the profession altogether both fell below the midpoint of 3, where lower scores are associated with lower turnover likelihood.

Table 3 includes Pearson’s correlations between intentions to leave and both health outcomes. PCS had non-significant relationships with turnover intention from either one’s role or profession altogether. MCS was weakly inversely correlated with both intentions to leave the role (r(283) = −0.23, *p* < 0.001) and intention to leave the profession (r(282) = −0.24, *p* < 0.001), indicating that lower mental health was associated with higher intention to leave within 12 months, but with small effect sizes.

### 3.4. Relationships Between Health, Job Demands, Job Resources, and Organisational Justice

Table 3 provides zero-order Pearson’s correlations between predictors and health outcomes. All Job Demands subscales and both Euthanasia Exposure measures had weak to moderate inverse correlations with both PCS and MCS, indicating that higher work pressure, cognitive demands, emotional demands, and direct and indirect euthanasia exposure were associated with poorer health across both physical and mental domains.

Among Job Resources subscales, social support was moderately positively correlated with both health outcomes. Autonomy and feedback were not significantly correlated with either. Greater social support was, therefore, associated with better health globally, and was the only studied job resource that produced a significant relationship with either outcome.

Distributive justice had moderate positive correlations with both PCS and MCS, whilst interpersonal justice had weaker significant positive relationships. Procedural justice was weakly positively correlated with MCS only. These results suggest that higher perceptions of the distributive justice dimension were the most strongly indicative of better health compared to the other OJ dimensions.

### 3.5. Predicting Health in Animal Care Workers in Shelter, Rescue, and Management Facilities

Variance in physical and mental health explained by demands and resources was assessed using hierarchical multiple regression analyses.

#### 3.5.1. Physical Health

In Table 4, age entered at step 1 explained 2.9% of the variance in PCS (R^2^ = 0.029, F(1166) = 5.02, *p* = 0.026). The addition of direct and indirect euthanasia exposure in step 2 explained an additional 7% of variance (R^2^ = 0.099, F(3164) = 6.02, *p* < 0.001). Work pressure, cognitive and emotional demands, and social support, entered at step 3, explained a further 11.1% of the variance in PCS (R^2^ = 0.210, F(7160) = 6.09, *p* < 0.001). Distributive and interpersonal justice at step 4 did not explain significant additional variance in PCS. The final model predicted 21.3% of the variance in PCS (R^2^ = 0.213, F(9158) = 4.75, *p* < 0.001). Increases in emotional demands and direct euthanasia exposure predicted reductions in PCS, while social support and age predicted increases in PCS.

#### 3.5.2. Mental Health

Table 5 shows a hierarchical regression with age entered in step 1 that explained 5.3% of the variance in MCS (R^2^ = 0.053, F(1166) = 9.21, *p* = 0.003). Entered at step 2, direct and indirect euthanasia exposure explained an additional 13.2% of variance (R^2^ = 0.185, F(3164) = 12.40, *p* < 0.001). Work pressure, cognitive demands, emotional demands, and social support at step 3 explained a further 23.1% of the variance in MCS (R^2^ = 0.416, F(7160) = 16.28, *p* < 0.001). OJ dimensions entered at step 4 did not significantly account for any further variance. The final model predicted approximately 42.4% of the total variance in MCS in the sample (R^2^ = 0.424, F(10,157) = 11.57, *p* < 0.001). Greater emotional demands and direct euthanasia exposure predicted decreases in MCS, whilst greater social support and age predicted increases.

## 4. Discussion

The present study aimed to explore job-related factors predicting global health in animal care workers working in animal management, shelters, and rescues, and how their health relates to intentions to leave.

### 4.1. Hypothesis 1: Global Health Characteristics of Animal Care Workers

The hypothesis that most participants would self-report below average physical and mental health was supported by the study findings. Results showed that, as a group, participants were below average for both mental and physical health when compared to the general population [80]. Further, a substantial proportion of participants were also below cutoff criteria for poor mental health (MCS) and physical health (PCS; [79]), indicating a higher likelihood of necessary healthcare usage or mortality in the future [81].

Although applications of these composites are limited amongst care workers, these results are notably lower than MCS and PCS means found in cancer care workers [67]. Findings are also consistent with previous literature that asserts animal care workers are vulnerable to impaired mental wellbeing [18,21,54], and they support suggestions that elements of these roles may have the capacity to impair physical health or induce physiological stress [19,34]. This indicates that physical health may require specific attention in this population in addition to the mental health and wellbeing factors already of concern.

### 4.2. Hypothesis 2: Animal Care Worker Health and Intentions to Leave

The hypothesis that animal care worker self-reported health would be inversely correlated with turnover intention was partially supported. Mental health had a significant, although weak, inverse relationship with both intentions to leave one’s role and one’s profession, but physical health was not significantly related to either.

Findings for mental health appear consistent with previous literature. Occupational stressors that can burden one’s mental health, such as secondary traumatic stress and burnout, have been linked with increases in turnover amongst animal care workers [54]. These findings taken together suggest that occupational stressors could eventually lead to declines in mental health that motivate intentions to leave.

Relationships to turnover may also be, in part, explained by the social satisfaction component of MCS. Those who work in animal care roles are notably passionate about their work, and many consider it a ‘calling’ [31]. Feelings of dedication to a role known for its high demands may be consuming, at the expense of external relationships. These social difficulties may in turn reduce job satisfaction and increase likelihood of turnover [82].

An unexpected finding was that physical health was not related to intentions to leave. This has several possible explanations. Poor physical health may be easier to make accommodations for at work, or at home, as compared to mental difficulties where more involved interventions [83] or reducing workload, and eventual turnover, may be necessary [33]. It is possible that some volunteers or part-time animal care workers take on opportunities in this field because they are either older or have an existing physical ailment that precludes them from regular full-time work. These individuals may persist in these roles despite such challenges due to satisfaction gained from the role [30] or the ease at which accommodation for these restrictions can be made, although additional research is required to explore this. Further, many animal care workers in shelters and rescues may work through their physical ailments on account of their empathy for animals in their care [30] or development of resilience [84]. It may also be that physical health decline is less recognisable because of this work, or those who have recognised their poor health have already left.

The sample overall reported a low likelihood of leaving the role or profession. This is surprising given the well-documented mental health challenges associated with these roles [17,21]. As mentioned, those experiencing significant health challenges may have already left, and those who remain may do so because they find the work rewarding [31] or because they can draw on resources to manage work demands. It is possible the present study captured a greater instance of those with reason or strategy to stay, as most participants had several years of experience.

### 4.3. Hypotheses 3 and 4: Occupational Predictors of Animal Care Worker Health and the Role of Organisational Justice

The findings supported the hypothesis that job demands would predict poorer self-reported health whilst higher resources would predict greater self-reported health. These findings support the relationships between demands, resources, and wellbeing outlined in the JD-R framework [50]. This did not apply to all demands and resources, however, as only emotional demands and social support predicted health across both mental and physical domains. Direct euthanasia exposure was also a significant predictor of poorer health for both domains. Indirect euthanasia exposure, however, was not a predictor for either outcome. An older age predicted better physical and mental health. While this was to be expected for mental health [85], the better perceived physical health was unexpected. It is unclear why older participants in this sample reported better physical health. Future research should determine whether older animal care workers have a lower expectation of good physical health and therefore perceive their physical health to be better than younger people, even if it might be objectively worse. The final hypothesis that higher OJ perceptions would predict greater health was not supported as it was not a significant predictor over and above other resources, for either mental or physical health domains.

#### 4.3.1. Emotional Demands

The finding that emotional demands accounted for the most notable negative variance in health, over and above work pressure and cognitive demands, was consistent with literature examining predictors of burnout and secondary traumatic stress in veterinary professionals [54], as well as of exhaustion in shelter workers [40]. Whilst the studied outcomes are often related to mental health or wellbeing, the findings of the present study imply that the effects of intangible demands, such as emotional demands, can affect physical health in this population as well. This may suggest reductions in physical health can occur because of how emotional demands affect other health domains, or that emotional stressors contribute to physiological heightening [34], such that resources for physical function are also depleted.

#### 4.3.2. Euthanasia Exposure

Direct euthanasia exposure as a significant predictor of mental health is consistent with suggestions that participation in euthanasia is distressing for animal care workers [15,24,86]. Interestingly, the opposite effect has previously been found in predicting traumatic stress in veterinarians, where indirect exposure was a significant predictor, whilst direct exposure was not [54]. This difference in findings could be explained by a large proportion of the present sample working in shelters, but not as veterinarians, where care workers in shelters or rescues may have less training or be less prepared [87], have less control over the decision [21], or the reason for euthanasia may conflict with one’s moral beliefs [35,36]. Further, these factors could explain why direct experience had a higher impact on health than indirect euthanasia exposure in this population. It is also possible that animal shelter and rescue workers may have personally acted as carers (e.g., foster carers) for the animals, in which the animals lived with the worker in their home before being euthanised. This could increase the emotional impact of euthanising the animals, but more research is needed to determine if this is a factor.

Direct euthanasia exposure significantly predicting physical health supports suggestions that physiological stress spikes can occur during the process [34] and extends this assertion by finding that there may be long term impacts on physical health. This may imply that amongst more tenured care workers, as in this sample, it is the repeated exposure over time that turns these temporarily alarmed states into more debilitating or recognisable physical ailments. Repeated cases of acute stress (i.e., chronic stress) like this can dysregulate autonomic function and over time lead to compromised immune, cardiovascular, and respiratory functions [88].

#### 4.3.3. Social Support

Of the resources measured, only social support from coworkers predicted health. This is consistent with previous findings that social support at work is an important protective resource for animal care workers [30,42,52], although it conflicts with Monaghan et al. [33] who found it was the least important variable associated with compassion fatigue, and Rohlf et al. [54], who reported similar. It is interesting that physical health was predicted by social support. As Rohlf et al. [40] described, productive workplace relationships may help animal care workers deal with the demands of their work, and thus the relationship between physical health and social support may be by proxy. Supportive colleagues might also notice physical difficulties before the individual themselves or encourage active breaks from work that promote physical health.

#### 4.3.4. Organisational Justice

The finding that no OJ dimension significantly predicted either health domain after accounting for other job resources was not anticipated. In other contexts, it has predicted various forms of wellbeing, work engagement, and psychological distress [57,59]. Similarly, in a previous study, worker beliefs that they are valued by their organisation predicted 17–25% of burnout variance in animal care workers [20]. It is possible that OJ did not produce a significant result as analyses were constructed to determine if it could predict variance in health over and above known demands and resources, rather than in isolation.

While OJ did not significantly predict health above other job resources, it was correlated with both physical and mental health. This may imply that OJ is related to factors that themselves predict these outcomes. For example, moral stress might be one mechanism ameliorated by OJ. Moral stress occurs when someone is required to act in a way that opposes their moral values [37], and moral injury is the subsequent emotional, psychological, behavioural, and social harm incurred by this [89]. Andrukonis and Protopopova [36] found those who participate in euthanasia in animal shelters have higher levels of moral injury, and this is related to their understanding of, and limited ability to input into, decisions made around the process. It is possible higher perceptions of OJ may reduce the instance of moral injury amongst workers and consequently lessen the impact of demanding tasks on animal shelter workers.

### 4.4. Theoretical and Practical Implications

The results of this study suggest that emotionally demanding tasks, participation in euthanasia, or other stressors not examined may stimulate broader decline in health than what has been examined in previous research of animal care workers. This decline may also encompass physical health and may do so regardless of whether the demand itself is physical. These findings give merit to exploring physical health as an outcome in this population.

This study was also the first to investigate OJ as a protective factor for animal care worker health. Findings indicated that, while OJ may not directly predict health after controlling for other more general demands and resources in animal care workers, they were correlated. This highlights the need for further investigation of OJ in this population, specifically whether it moderates the relationship between certain demands, such as moral stress and health. Understanding organisation-oriented factors like OJ may inform proactive strategies to prevent poor outcomes from occupational stress.

Managers in organisations could better understand the impact of occupational stressors on global health by monitoring the mental and physical health of workers through regular, voluntary, and confidential routine records, as is done in other high-stress roles like emergency responders [90]. This may help monitor decline; inform necessary accommodations to minimise turnover; and raise awareness for how health difficulties may result from cumulative or repeated stressors, rather than just isolated events. In innately high-stress roles, this kind of workers’ health surveillance is used to inform earlier interventions that support an individual’s health and continued work [90].

Employers in industries with inherent or unavoidable high stresses, as with those in animal shelters, management, and rescues, may also consider rotating employee tasks. Whilst this is noted to improve job satisfaction, psychological health, and physical health [91], it may also lessen the frequency of engagement with demanding tasks.

### 4.5. Limitations and Future Research

A limitation of this study is that mental and physical health can influence one another [92]. This creates challenges when determining if a demand or resource impacts a health domain in a unique way, or if this results from a change in another domain. For this study, comparison of two similar and simple scales of mental and physical health was used to assess if there were any overt differences in how demands and resources predicted these in animal care workers. However, as predictors were similar between health outcomes, future research exploring factors influencing physical health should consider controlling for the impact of mental health. Future studies of physical health amongst animal care workers are also encouraged, as it is evident that this can be negatively affected by this work and may benefit from unique supports not examined here.

Further, this study had an experienced sample with several years in the animal care work in the sector. This limits definitive conclusions regarding turnover, due to significant turnover occurring early in career [42]. Additionally, there may be shared traits between experienced participants, such as resilience [84], that influence their perception of work and health that then impact self-report scales used in this study. Future studies should attempt to control for these interpersonal similarities and differences through longitudinal research that captures change in perceptions of stress, health, and intentions to leave reported by participants over time.

## 5. Conclusions

The present study aimed to determine whether animal care work in management, sheltering, and rescue can impact global health by exploring how job-related factors such as demands and resources were related, including the role of organisational justice, using the JD-R model. Further, this study also aimed to explore the nature of any relationship between global health in this population, as well as intentions to leave one’s role or the industry altogether. Below average mental and physical health supported existing findings regarding mental wellbeing and added physical health as a new area of impact. Emotional demands, direct euthanasia exposure, and social support were revealed as significant predictors of mental and physical health alike, highlighting areas where these health domains are similarly impacted, as well as how physical health can be afflicted by intangible demands. Organisational justice was not predictive of health, but it was correlated.

The present study adopted a broader definition of health than just that which is linked to mental wellbeing, as has previously dominated research within this population and research area. Through this study it is apparent that elements of physical health may also be impacted in these roles, which may occur as a result of changes in mental health or may be affected independently and be in need of further research. Further, to the authors’ knowledge, this study was the first to examine organisational justice as a resource for animal care workers, although this requires additional inquiry from future research.

The findings of this study may suggest a lack of emphasis on health in the animal care industry, possibly due to either low awareness or inadequate knowledge of the ways in which health may be impacted by work-related stressors. Those in these fields should consider how the temporary and targeted wellbeing impact of high stress events may lead to more stable or broad health decline and take ongoing record of health to better identify point of decline to inform supports.

Future research would benefit from analysis of demands or resources uniquely related to physical health to explain if declines in this domain are isolated or a product of declines in mental health. Further, additional research is required to determine if organisational justice serves a moderating role between job demands and health outcomes.

## Figures and Tables

**Table 1:** Demographic characteristics of participants working in animal shelter, rescue, and management.

	Demographic	*N*	%
Gender (*N* = 213)	Female	189	88.7
	Male	19	8.9
	Non-binary	13	1.4
	Prefer not to say	2	0.9
Employment location (*N* = 285)	Suburb	128	44.9
	Regional	66	23.2
	City	58	20.4
	Rural	33	11.6
Employment community SES (*N* = 284)	Mixed	164	57.7
	Medium	84	29.6
	Low	34	12.0
	High	2	0.7
Organisation type (*N* = 285)	Volunteer-run rescue	131	46.0
	NFP shelter	110	38.6
	LG facility	38	13.3
	Veterinary clinic	6	2.1
Participant time in sector (*N* = 284)	<1 year	21	7.4
	1–2 years	50	17.6
	3–5 years	56	19.7
	6–10 years	56	19.7
	>10 years	101	35.6
Paid an income (*N* = 285)	Yes	87	30.5
	No	198	69.5
Role ^1^ in organisation (*N* = 285)	Management/leadership	148	51.9
	Volunteer	154	54.0
	General animal care	131	45.9
	Administration	116	40.7
	Foster carer	132	46.3
	Transport	76	26.7
	Ranger	7	2.5

Note: SES = socioeconomic status. NFP = not-for-profit. LG = local government. ^1^ Participants could select more than one role; thus, percentage figures are of the total sample, *N* = 285.

**Table 2:** Sample size, means, standard deviations, and Cronbach’s alphas of the PROMIS-GH, intentions to leave, job demands, exposure to euthanasia, job resources, and organisational justice.

Variable		*N*	Cronbach’s α	M	SD	Min ^1^	Max ^2^
PROMIS-GH	PCS t-score	285	0.74	43.9	7.7	19.9	61.9
	MCS t-score	285	0.85	42.1	9.1	21.2	67.6
Intention to leave	Current role	285	-	1.9	1.3	1.0	5.0
	Profession	284	-	1.8	1.1	1.0	5.0
Job demands	Overall	285	0.94	3.4	1.0	1.0	5.0
	Work pressure	284	0.92	3.3	1.3	1.0	5.0
	Cognitive demands	282	0.88	3.4	1.1	1.0	5.0
	Emotional demands	285	0.87	3.7	1.1	1.3	5.0
Euthanasia exposure	Direct	226	-	2.6	1.7	1.0	5.0
	Indirect	226	-	3.5	1.5	1.0	5.0
Job resources	Overall	285	0.89	3.4	0.9	1.0	5.0
	Social support	233	0.88	3.7	1.1	1.3	5.0
	Autonomy	285	0.82	3.6	1.1	1.0	5.0
	Feedback	284	0.89	2.9	1.1	1.0	5.0
Organisational justice	Overall	285	0.85	3.7	0.8	1.0	5.0
	Distributive	285	0.89	3.3	1.1	1.0	5.0
	Procedural	279	0.83	3.7	1.0	1.0	5.0
	Interpersonal	276	0.92	4.1	1.0	1.0	5.0

Note. PROMIS-GH = Patient Reported Outcomes Measurement Information System—Global Health. PCS = physical composite score. MCS = mental composite score. ^1^ Possible minimum values; PCS = 19.9. MCS = 21.2. All other subscales = 1. ^2^ Possible maximum values; PCS = 61.9. MCS = 67.6. All other subscales = 5.

**Table 3:** Person correlations for physical and mental health outcomes against intentions to leave, organisational justice, job demands, job resources, exposure to euthanasia, and age.

Variable		Physical Composite Score	Mental Composite Score
Intentions to leave	Role	−0.09	−0.23 ***
	Profession	−0.10	−0.24 ***
Organisational justice	Overall	0.13 *	0.28 ***
	Distributive	0.25 ***	0.37 ***
	Procedural	−0.03	0.12 *
	Interpersonal	0.13 *	0.18 **
Job demands	Overall	−0.31 ***	−0.44 ***
	Work pressure	−0.25 ***	−0.34 ***
	Cognitive demands	−0.27 ***	−0.38 ***
	Emotional demands	−0.35 ***	−0.50 ***
Job resources	Overall	0.06	0.18 **
	Autonomy	0.01	0.11
	Social support	0.26 ***	0.39 ***
	Feedback	0.01	0.12
Euthanasia exposure	Direct	−0.26 ***	−0.38 ***
	Indirect	−0.15 *	−0.23 ***
Age		0.17 *	0.23 ***

Note: * *p* < 0.05, two-tailed. ** *p* < 0.01, two-tailed. *** *p* < 0.001, two-tailed.

**Table 4:** Hierarchical multiple regression predicting physical health in animal care workers from job demands, job resources, and organisational justice.

	Predictors	Physical Composite Score					
		R^2^	ΔR^2^	*B*	*SE*	β	*p*
Step 1		0.03	0.03 *				
	Age			0.09	0.04	0.17 *	0.026
Step 2		0.10	0.07 **				
	Age			0.04	0.04	0.17 *	0.021
	Euthanasia exposure (direct)			−1.22	0.39	−0.26 **	0.002
	Euthanasia exposure (indirect)			−0.04	0.43	−0.01	0.923
Step 3		0.21	0.11 ***				
	Age			0.09	0.04	0.17 *	0.019
	Euthanasia exposure (direct)			−0.82	0.41	−0.18 *	0.046
	Euthanasia exposure (indirect)			0.22	0.43	0.04	0.608
	Work pressure			0.00	0.76	0.00	0.997
	Cognitive demands			0.63	0.93	0.09	0.500
	Emotional demands			−2.08	0.71	−0.30 **	0.004
	Social support			1.65	0.53	0.23 **	0.002
Step 4		0.21	0.00				
	Age			0.09	0.04	0.17 *	0.020
	Euthanasia exposure (direct)			−0.82	0.41	−0.18 *	0.047
	Euthanasia exposure (indirect)			0.24	0.44	0.05	0.582
	Work pressure			0.05	0.77	0.01	0.945
	Cognitive demands			0.65	0.94	0.09	0.487
	Emotional demands			−2.01	0.72	−0.29 **	0.006
	Social support			1.65	0.61	0.23 **	0.007
	Distributive justice			0.38	0.60	0.05	0.524
	Interpersonal justice			−0.24	0.65	−0.03	0.717

Note: * *p* < 0.05, two-tailed. ** *p* < 0.01, two-tailed. *** *p* < 0.001, two-tailed.

**Table 5:** Hierarchical multiple regression predicting mental health in animal care workers from job demands, job resources, and organisational justice.

	Predictors	Mental Composite Score					
		R^2^	ΔR^2^	*B*	*SE*	β	*p*
Step 1		0.05	0.5 **				
	Age			0.13	0.04	0.23 **	0.003
Step 2		0.19	0.13 ***				
	Age			0.13	0.04	0.23 **	0.002
	Euthanasia exposure (direct)			−1.87	0.43	−0.34 ***	<0.001
	Euthanasia exposure (indirect)			−0.29	0.48	−0.05	0.556
Step 3		0.42	0.23 ***				
	Age			0.13	0.04	0.23 ***	<0.001
	Euthanasia exposure (direct)			−1.21	0.41	−0.22 **	0.004
	Euthanasia exposure (indirect)			0.12	0.44	0.02	0.790
	Work pressure			0.23	0.77	0.03	0.766
	Cognitive demands			0.80	0.94	0.10	0.396
	Emotional demands			−3.40	0.71	−0.41 ***	<0.001
	Social support			2.91	0.53	0.34 ***	<0.001
Step 4		0.42	0.01				
	Age			0.13	0.04	0.23 ***	<0.001
	Euthanasia exposure (direct)			−1.16	0.42	−0.21 **	0.006
	Euthanasia exposure (indirect)			0.14	0.44	0.02	0.745
	Work pressure			0.39	0.78	−0.05	0.622
	Cognitive demands			0.79	0.95	0.10	0.404
	Emotional demands			−3.24	0.73	−0.39 ***	<0.001
	Social support			3.00	0.62	0.35 ***	<0.001
	Distributive justice			0.82	0.62	0.10	0.185
	Procedural justice			−0.45	0.72	−0.05	0.536
	Interpersonal justice			−0.34	0.72	0.04	0.641

Note: ** *p* < 0.01, two-tailed. *** *p* < 0.001, two-tailed.

## Data Availability

Data are unavailable due to privacy restrictions.
